# Host mRNA Analysis of Periodontal Disease Patients Positive for *Porphyromonas gingivalis*, *Aggregatibacter actinomycetemcomitans* and *Tannerella forsythia*

**DOI:** 10.3390/ijms23179915

**Published:** 2022-08-31

**Authors:** Ramona Gabriela Ursu, Luminita Smaranda Iancu, Elena Porumb-Andrese, Costin Damian, Roxana Gabriela Cobzaru, Giorgio Nichitean, Carmen Ripa, Darius Sandu, Ionut Luchian

**Affiliations:** 1 Department of Preventive Medicine and Interdisciplinarity (IX)—Discipline of Microbiology, Faculty of Medicine, “Grigore T. Popa” University of Medicine and Pharmacy, 700115 Iasi, Romania; 2 Department of Medical Specialties (III)—Discipline of Dermatology, Faculty of Medicine, “Grigore T. Popa” University of Medicine and Pharmacy, 700115 Iasi, Romania; 3 Faculty of Dental Medicine, “Grigore T. Popa” University of Medicine and Pharmacy, 700115 Iasi, Romania; 4 Department of Periodontology, Faculty of Dental Medicine, “Grigore T. Popa” University of Medicine and Pharmacy, 700115 Iasi, Romania

**Keywords:** biomarkers, early detection, cell lines, animal model, target therapy, periodontal disease, mRNA, periodontitis

## Abstract

Periodontal disease is a frequent pathology worldwide, with a constantly increasing prevalence. For the optimal management of periodontal disease, there is a need to take advantage of actual technology to understand the bacterial etiology correlated with the pathogenic mechanisms, risk factors and treatment protocols. We analyzed the scientific literature published in the last 5 years regarding the recent applications of mRNA analysis in periodontal disease for the main known bacterial species considered to be the etiological agents: *Porphyromonas gingivalis*, *Aggregatibacter actinomycetemcomitans* and *Tannerella forsythia.* We identified new pathogenic mechanisms, therapeutic target genes and possible pathways to prevent periodontal disease. The mRNA analysis, as well as the important technological progress in recent years, supports its implementation in the routine management of periodontal disease patients.

## 1. Introduction

**Figure 1:**
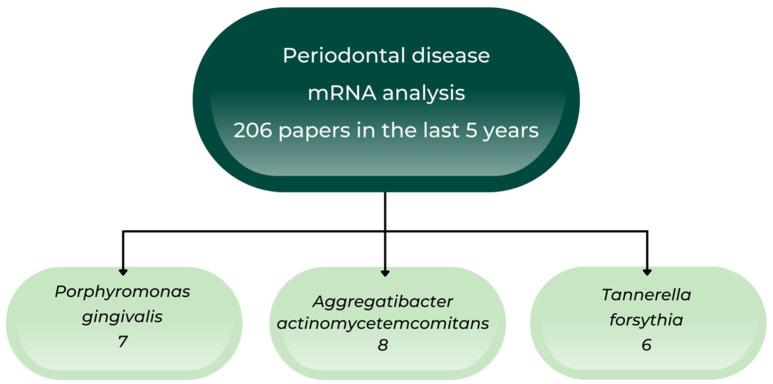
Diagram of analyzed studies.

The pathogenic mechanism of periodontal disease is a complex interaction between plaque bacteria and a susceptible host, characterized by an inflammatory process that leads to the destruction of attachment tissues and bone loss [1]. It is a widespread disease, with approximately half of people over 30 being affected [1], while the severe form affects approximately 11% of the global population and poses a high burden on healthcare systems, generating costs that reach billions of dollars each year. This is why there is a high need to find novel diagnostic assays and to better understand the pathogenic mechanisms [2]. Thanks to data gathered from carefully conducted, longitudinal monitoring studies, the understanding of the prevalence of periodontal disease has changed. The pathogenic mechanisms are now better understood, and this change has led to a shift from an older to a more recent theory. The old theory of periodontal disease was that periodontitis is an inevitable consequence of gingivitis, that it is uniformly distributed in the population, that disease severity is correlated with plaque levels, which lead to a linear, progressive loss of attachment over time, and that the severity of periodontitis increases with age. By comparison, the new theory of periodontitis states that gingivitis and mild periodontitis are common (seen in about 40–60% of people), and approximately 10–15% of the population exhibit advanced periodontitis. Gingivitis precedes periodontitis, but not all sites with gingivitis develop periodontitis, and periodontitis is not a natural consequence of aging. The most frequent periodontal pathogens recognized in 2004 were Gram-negative species such as *Actinobacillus actinomycetemcomitans* (currently *Aggregatibacter actinomycetemcomitans*), *Porphyromonas gingivalis*, *Bacteroides forsythus* (currently *Tannerella forsythia*) and *Eikenella corrodens* [3]. The concepts of periodontal disease from 2004 were recently updated by Kwon, T. et al., 2021, who recognized that the prevalence of periodontal disease increased from 15% to more than 40% in American adults. The authors also note the multifactorial etiology of periodontal disease, including subgingival dental biofilm [4]. The disease pathogenesis is presented in Figure 1.

Bringuier, A. et al. detected *Methanobrevibacter oralis* by qRT PCR in both periodontal patients and age-matched controls, at similar proportions (100% vs. 80%) [5].

Due to methanogen-targeting molecular investigations, the oral cavity inhabitant, *Methanobrevibacter oralis*, was found to be more strongly associated with periodontitis pockets in association with anaerobes, and its role was analyzed as moderate [6]. 

A comprehensive, recent review presents the roles of normal flora from the oral cavity, and mentions its function in oral mucosa homeostasis and in stimulating the immune system, especially in the case of periodontitis [7].

Periodontal disease management requires standardization in the diagnosis and reporting of chronic periodontitis. A multidisciplinary team underlined the need to note the study design (e.g., inclusion criteria for participants, regional versus national study, type of sampling, sample size), assess periodontal measurements and record the protocols (e.g., analyzing periodontal pockets, probing pocket depth, a full-mouth recording), the need for a periodontal probe (inter-and intra-examiner variability is very important), and the importance of ensuring the examiners’ reliability.

The authors referred to the manner of reporting periodontal studies regarding the characteristics of study subjects, and the reporting of the prevalence and severity of periodontal diseases in accordance with periodontal case definitions and gingival inflammation. All these determinants of periodontitis prevalence and severity can optimize the control of the burden of periodontitis worldwide. By using standardized protocols when reporting each case of periodontal disease, variations between different populations will be eliminated [8].

In an observational study, another international research team (Germany, Hong Kong, and Spain) underlined the need for standardization in periodontal disease screening. The authors compared specific databases and found that bleeding on probing has the strongest association with severe periodontitis. They developed an easy-to-use guide for daily practice [9].

As in other medical fields, in periodontal disease, Swedish researchers implemented a means of monitoring dental health and healthcare by registering data about patients (gender, age, living area, dental status, risk assessments for caries and periodontitis and dental care provided). This systemic registration of oral health and quality of dental care will facilitate clinical and epidemiological research and randomized controlled trials [10]. In Sweden, a research team performed a longitudinal study regarding indicators of periodontitis, such as alveolar bone loss, for ten years in an older population. The authors used a questionnaire and performed a clinical examination. Alveolar bone loss was associated with poor general health and irregularly undergoing dental care [11]. Periodontal disease was found to be associated with other pathologies, such as rheumatoid arthritis [12] and asymptomatic carotid plaque [13].

Although the main scientific databases contain many recent papers regarding the identification of periodontal pathogens, we only found 206 articles published in the last five years referring to the importance of mRNA analysis in periodontal diagnosis (Figure 1).

The aim of this review was to analyze the scientific literature published in the last five years regarding the recent applications of mRNA analysis to periodontal disease for the main known bacterial species considered to be the etiological agents: *Porphyromonas gingivalis*, *Aggregatibacter actinomycetemcomitans*, and *Tannerella forsythia*. We referred to circular RNAs in periodontal disease, and to the actual issues and controversies regarding the optimal therapy of periodontal diseases.

## 2. Updates in Periodontal Disease Classification

In 2018, a consensus report was published on the classification of periodontal and peri-implant diseases and conditions. This update was necessary because, in recent years, the classification of periodontitis has been repeatedly modified in an extremely important attempt to align it with the newest scientific evidence [14].

**Figure 2:**
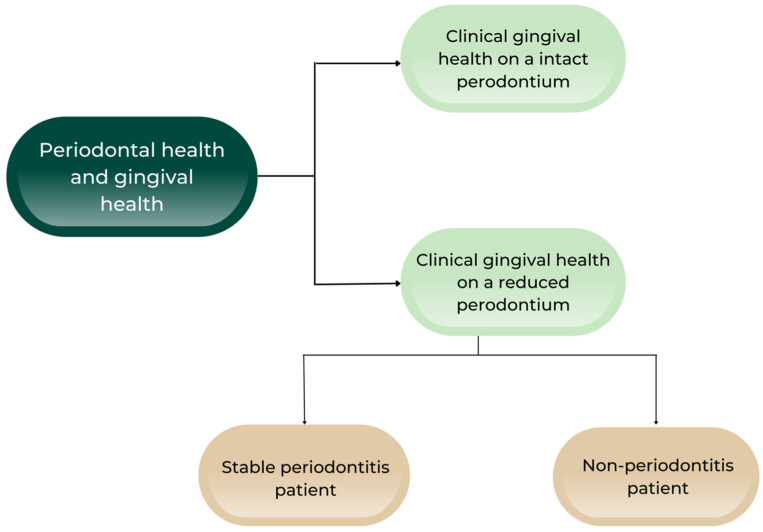
Periodontal and gingival health [14].

In order to provide a properly updated version of the previous classification of Armitage, it was mandatory for the members of the study group to redefine the state of periodontal and gingival health (Figure 2).

Jepsen S et al. 2018, provided an updated classification of the periodontal manifestations and conditions affecting the course of periodontitis and the periodontal attachment apparatus, as well as its development and acquired conditions. The authors presented the systemic diseases and conditions that affect the periodontal supporting tissues, e.g., diseases associated with immunologic disorders such as Down syndrome, acquired immunodeficiency diseases (e.g., acquired neutropenia), inflammatory diseases such as *Epidermolysis bullosa acquisita*, other systemic disorders that influence the pathogenesis of periodontal diseases, such as diabetes mellitus, and neoplasms that can result in the loss of periodontal tissues independent of periodontitis. Mucogingival conditions related to natural dentition referred to possible consequences of gingival recession and root surface exposure to oral environment, the development of gingival recession associated with the gingival phenotype, periodontal phenotype assessments in a standardized and reproducible way and the classification of gingival recession [15].

**Figure 3:**
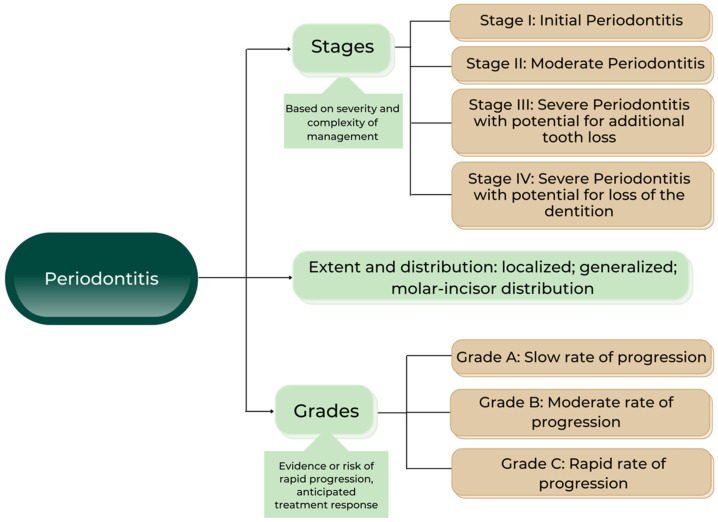
Classification of periodontitis [14].

The current classification introduced a much more accurate and predictable approach to the diagnosis of periodontitis, using stages and grades and referring to the extent and distribution. Stages are based on the severity and complexity of management, while grades refer to evidence or risk or progression in the context of anticipation of the treatment response (Figure 3).

The recently published clinical practice guidelines for the treatment of periodontitis in stages I–III provided evidence-based recommendations for the treatment of periodontitis patients, defined according to the 2018 classification [16]. Stage IV periodontitis was recently updated to maintain a healthy dentition over one’s lifetime [17]. 

## 3. Biomarkers Early Detection by mRNA Assays

MicroRNA was reviewed by Catalanotto C et al. and the authors mentioned its influence on many physiological processes, such as differentiation, proliferation, apoptosis and development, its cytoplasmatic functions, nuclear functions and host cell’s miRNAs, which target viral mRNAs [18]. To analyze the RNA, there is a need for cutting-edge technologies in the field of molecular biology to implement novel biotechnological and medical applications of RNA, such as, for example, in regenerative medicine to promote stronger cardiovascular outcomes [19]. Several mRNA methods have been used in the management of other diseases, such as in the case of non-small lung cell carcinoma, where a research team used a large array of such investigations: Western Blot analysis and antibodies detection, protein extracts from human tissue samples, cell Cultures, siRNA and DNA transfections, plasmids and cloning strategies, total mRNA extracts’ purification, RT-qPCR and RT-ddPCR analysis, RNA immunoprecipitation and RNA chromatography assays, migration, invasion and proliferation assays, flow-cytometry analysis, epifluorescence microscopy and immunohistochemistry [20].

circRNAs exhibit specific characteristics, making them ideal biomarkers for diagnosis and prognosis. Mi Z et al. mentioned in a review that traditional methods, such as northern blotting, RT-qPCR and microarray analysis, provide useful but limited information. New techniques are available for circRNA detection, such as RT-ddPCR, RCA and LAMP, with their own advantages and limitations [21]. A recent review (September 2022) mentions the latest developments in the analysis of nucleic acids using capillary electrophoresis and its applications for ASO, siRNA, mRNA, gRNA, microRNA, AAV and aptamers. Each of these therapeutic nucleic acid analyses should be tested in terms of analytical challenges and future perspectives [22].

In Table 1, the recent findings in periodontal disease using mRNA analysis for *Porphyromonas gingivalis* are presented. The studies were performed from Finland to China; the authors used samples from patients (e.g., gingival biopsies) and studied different in vitro models (e.g., cell lines). The findings were especially correlated with the identification of different molecules by mRNA, which could elucidate the pathogenesis of periodontal disease: caspase-4 activation in *P. gingivalis*-infected gingival epithelial cells (GECs), monocyte chemoattractant protein-1-induced protein (MCPIP-1) and mucosa-associated lymphoid tissue lymphoma translocation protein (MALT-1) responses, the target gene MZB1, CTHRC1. Type IX protein secretion system (T9SS) shutdown was found to influence the inflammatory response in periodontal pathogens and is also considered a potential novel target for periodontal therapy. 

The analyzed studies referring to periodontal disease and *Aggregatibacter actinomycetemcomitans* used an in vivo model (rats and mice), cell lines, plant extracts and human tissues. Using mRNA analysis, the authors found that bone resorption and osteoclast genesis can be influenced by different therapies for chronic periodontitis. The plant extracts were candidates for oral hygiene products to optimize periodontal health. Other studies analyzed different substances with anti-inflammatory activities in periodontal patients. We identified some very interesting associations between periodontal disease and other pathologies, such as Alzheimer’s disease and rheumatoid arthritis (Table 2). 

The mRNA analysis regarding *Tannerella forsythia* mainly tried to identify pathogenic mechanisms (KLIKK-proteases, cytokine IL-1α levels, NLRP3 and AIM2 proteins, and TREM-1 (triggering receptor expressed on myeloid cells 1) tissue expression) and preventive actions (*Litsea japonica* leaf extract) (Table 3).

## 4. Oral Anaerobic Bacteria and Cancer

*Prevotella*, *Fusobacterium*, *Porphyromonas*, *Treponema* and *Aggregatibacter* genera were associated with periodontal disease in a recent review [7].

While the most important and well-studied human pathogens associated with cancers are viruses [43], in recent years, a link has begun to appear between anaerobic bacteria found in the oral cavity and tumors of the gastrointestinal tract, especially colorectal carcinoma, with the most important representative being *Fusobacterium nucleatum*. This is a Gram-negative, anaerobic bacteria that can be found as part of the oral microbiome, and when dysbiosis occurs in association with other bacteria, it produces gingivitis and periodontal disease. *F. nucleatum* seems to tend to disseminate from the oral cavity to other sites of the human body, probably via hematogenous transfer, and is frequently found in placental and fetal tissues, especially in adverse pregnancy outcomes [44].

While abundant in the oral microbiota, this bacterium is seldom found in the healthy colon, although numerous studies have shown an increased presence of *F. nucleatum* in colorectal cancer samples. Castellarin et al., as well as Kostic et al., found increased levels of fusobacterial nucleic acids in colorectal carcinoma samples in 2012, while the same increase was not present in adjacent normal tissues [45,46]. Since *F. nucleatum* is not normally present in high amounts in the lower GI tract, it has been proposed that the presence of this bacteria is used in colorectal carcinoma screening, diagnosis and disease follow-up. In a meta-analysis study, Zhang et al. found that testing for *F. nucleatum* alone in fecal samples has a pooled sensitivity and specificity of 71 and 76%, respectively, making it a valuable tool in the diagnosis of such tumors [47]. Guo et al. compared the ratios of *F. nucleatum* to other bacteria normally present in fecal samples (*Faecalibacterium prausnitzii*, *Bifidobacterium* spp., *Lactobacillus*) in two cohorts composed of 903 patients, and found the *F. nucleatum/Bifidobacterium* ratio to have an 84.6% specificity and 92.3% sensitivity in detecting colorectal carcinoma. Combining *F. nucleatum/Bifidobacterium* with *F. nucleatum/F. prausnitzii* assays could be an efficient, noninvasive screening test, able to detect stage I colorectal carcinoma with 60% specificity and 90% sensitivity [48]. Other authors proposed wider detection panels, using *Parvimonas micra*, *Peptostreptococcus stomatis*, *Fusobacterium nucleatum* and *Akkermansia muciniphila* as cancer biomarkers [49]. 

The specific target used by *F. nucleatum* to recognize and adhere to tumoral cells is the molecule d-galactose-β(1-3)-*N*-acetyl-d-galactosamine, commonly known as Gal/GalNAc, which is overly abundant in colorectal carcinoma. The specific ligand for Gal/GalNAc seems to be the fusobacterial adhesin Fap2 [50], an important virulence factor of *F. nucleatum*, especially for co-aggregation, together with other oral anaerobes such as *Porphyromonas gingivalis* in the pathogenic mechanism of periodontitis [51]. Placental tissues are also rich in Gal/GalNAc; thus, the mechanism of *F. nucleatum* colonization of the placenta in adverse pregnancy outcomes must be due to the same Gal/GaNAc-Fap2 interaction [52].

Another type of tumor that is particularly abundant in Gal/GalNAc is breast cancer, and the level of this marker increases with tumoral progression, a discovery that can now explain the high prevalence of *F. nucleatum* in the breast cancer microbiome [53]. Experimental studies in mouse models showed that, in addition to colonizing the tumoral tissues, *F. nucleatum* has negative effects on disease progression and metastatic development, inducing the suppression of T-cell numbers in the tumor. By intravenously inoculating one group of mice with *F. nucleatum* capable of expressing Fap2, and another group with a Fap2-deficient strain, the authors showed that Fap2 is vital for tumor colonization, and that tumors colonized with these anaerobic bacteria had an increased size and number of metastases [53].

Regarding therapy outcomes and survival, Kunzmann et al. found that although high *F. nucleatum* DNA levels in colorectal tumors are associated with poorer survival outcomes, their study indicated that this assay has limited clinical use for predicting prognosis [54]. In esophageal carcinoma, some authors found that high levels of intratumoral *F. nucleatum* are significant when predicting a poorer response to neoadjuvant chemotherapy and suggest that antibiotic therapy could improve outcomes [55].

While some authors focus on antibiotics as a solution to combat periodontitis pathogens in the oral cavity [56], some novel therapeutic strategies have also been proposed in studies that link those pathogens to cancer. One such strategy, which exploits the association between *Fusobacterium nucleatum* and colorectal carcinoma, has been studied by Zheng et al. using a nanotechnological microbiome-modulating intervention. The research team used a bacteriophage that specifically targets *F. nucleatum* to reach tumoral tissues and lyse these bacteria, reducing their pro-tumoral effects [57].

## 5. Circular RNAs Assessing in Periodontal Disease

Circular RNAs (circRNAs) can influence disease progression by targeting miRNA/mRNA axis. Periodontal disease was intensely studied in connection with this biomarker. Deng W et al. used different assays (qRT-PCR, cell proliferation, wound healing, cell apoptosis, enzyme-linked immunosorbent assay (ELISA)) on periodontitis cell models and identified that circ_0138959 was overexpressed in periodontitis tissues and LPS-treated periodontal ligament cells, which could be a suitable target for periodontal disease therapy [58].

Another possible therapeutic target for periodontal disease is circ_0062491, which was found by Wang L et al. to protect PDLCs from LPS-induced apoptosis and inflammation. In this study, the authors used cell counting Kit-8 (CCK-8) assay, flow cytometry and Western blot, in addition to the above-mentioned techniques [59]. Using the previously mentioned assays, together with the dual-luciferase reporter assay and RNA immunoprecipitation assay for validation of target interaction, Li Q et al. showed that circ_0066881 partly prevented LPS-evoked cell dysfunction in PDLCs through the miR-144-5p-mediated up-regulation of retinoid acid-related orphan receptor A [60].

Using high-throughput sequencing and qRT-PCR, Yu W et al. identified differentially expressed circRNAs in gingival tissues from periodontitis patients, as it is known that periodontal disease is a chronic multifactorial inflammatory disease. The authors detected 70 differentially expressed circRNAs (68 up-regulated and 2 down-regulated circRNAs) in human periodontitis, and they found a positive correlation between up-regulated circRNAs, circPTP4A2, chr22:23101560-23135351+, circARHGEF28, circBARD1 and circRASA2, and the PD-suggested function of circRNAs in periodontitis [61].

A very interesting study analyzed the circRNAs in periodontal tissues in patients with or without *Redondoviridae*-infection—DNA viruses known to be associated with periodontitis. The authors used a high-throughput RNA sequencing assay to understand the pathogenetic mechanisms of the *Redondoviridae*-related periodontitis, to see if it is possible to use these viruses as biomarkers and, in future, targeted therapies [62]. 

Circular RNAs’ role in periodontal disease has also been studied by other authors, with the main findings being the overexpression of hsa_circ_0003948 with a protective effect in chronic periodontitis via miR-144-3p/NR2F2/PTEN signaling regulation [63], and a promising biomarker for periodontitis treatment, circ_0085289, alleviated PDLC injury induced by LPS stimulation by modulating the let-7f-5p/SOCS6 axis [64].

circRNAs are starting to have more applications in periodontal disease, as they can be used to accurately diagnose periodontitis activity: circRNAs are expressed in periodontal cells in a cell-specific manner, can function as microRNA sponges and can form circRNA-miRNA–mRNA networks during osteogenic differentiation for periodontal-tissue (or dental pulp)-derived progenitor cells [65]. The above-mentioned studies underline the opportunity revealed by RNA analysis in periodontal diseases, which could lead to an understanding of the pathogenetic mechanisms and to new targeted therapies.

## 6. RNA-seq and Periodontal Diseases

Teles F et al. recognized the utility of RNA sequencing in periodontal disease, in a recent review. The authors found new NGS findings regarding the relationship between periodontal disease and systemic factors, with benefits for the patient for follow-up and therapy [66].

There are many published studies regarding periodontal disease and RNA sequencing analysis. Chen X et al. used 16S rRNA gene sequencing analyses for patients with Crohn’s disease and found that both red complex (*Porphyromonas*, *Tannerella* and *Treponema*) and orange complex (*Fusobacteria*) bacteria were abundant in periodontitis subgingival plaque, in comparison with orange complex bacteria (*Prevotella*_2 and *Prevotella*), which was overexpressed in Crohn’s disease-associated periodontitis subgingival plaque. The authors recognize the advantage of using 16S rRNA to reveal the oral microbiome in CD-associated periodontitis in comparison with periodontal patients without this condition [67].

Ge D et al. used the 16S rRNA sequence of *P. gingivalis* for studying patients with periodontal disease. The recombinase polymerase amplification, combined with nanoparticle-based lateral flow strips for the rapid detection of *P. gingivalis*, was found to be an efficient, rapid (30 min) and convenient diagnostic method that optimizes the classical diagnosis of detecting *P. gingivalis* [68].

In a recent meta-analysis, Jiang Y et al. made a comparison between saliva and subgingival plaque using 16S rRNA gene sequencing techniques. They revealed that both the detection frequencies and relative abundances of red-complex bacteria in saliva were significantly lower than those in subgingival plaque, leading to the conclusion that there is a need for further longitudinal clinical studies to evaluate the role of saliva [69]. 

Using 16S rRNA amplicon sequencing, Chang C et al. analyzed the relationship between periodontal pathogens and oral squamous cell carcinoma (OSCC). The researchers identified that *P. gingivalis* and *F. nucleatum* were present at higher levels in cancer tissue than in normal tissues and were correlated with subgingival plaques. This raised awareness regarding the involvement of the above periodontal pathogens and OSCC, in addition to the known risk factors, such as HPV, smoking and chronic alcohol use [70,71].

Smoking, as a risk factor altering salivary microbiomes, was analyzed in a prospective study using sequencing of 16S recombinant RNA gene amplicons. It is important to mention that *Porphyromonas gingivalis* was significantly more abundant in smokers, which suggests that smoking could influence the salivary microbiome and affect marginal bone loss during bone healing [72].

The microbial 16S rRNA gene sequencing was performed by Lundmark A. et al. to assess whether salivary microbiota is associated with host inflammatory mediators in periodontitis. The Swedish authors identified distinct and disease-specific patterns of salivary microbial composition between patients with periodontitis and healthy controls, noting that *Tannerella forsythia* was more frequently present in periodontitis [73].

Moreno C et al. conducted a meta-analysis that included two independent RNA-seq datasets to identify diagnostic biomarkers and specific pathways for a new targeted periodontitis therapy, such as chronic inflammation. The authors compiled a list of the top 10 drugs that should be further tested for their efficacy in treating periodontitis [74]. 

All the above-mentioned studies underline the clinical utility of RNA-seq in different clinical conditions associated with periodontal diseases. 

## 7. The Optimal Therapy of Periodontal Diseases

We analyzed recent randomized controlled trials regarding the antibiotic therapy of periodontal disease.

Blanco C et al. studied the clinical, radiographic and microbiological outcomes after non-surgical therapy of peri-implantitis for 32 patients, followed up for a period of 12 months. The authors considered that metronidazole as a systemic therapy led to significant additional improvements in clinical, radiographic and microbiological parameters [75].

Teles FRF et al. studied the percentage and taxonomy of minocycline-resistant isolates in saliva and subgingival plaque samples before and after minocycline microspheres application in periodontitis patients during maintenance. The patients were monitored for 6 months, and the authors found that even *Aggregatibacter actinomycetemcomitans*, *Tannerella forsythia* and *Porphyromonas gingivalis* were sensitive to antibiotics, and minocycline microspheres resulted in the transient selection of minocycline resistant species in saliva and subgingival plaque samples, such as *Gemella morbillorum* and *Eubacterium saburreum* [76].

Cosgarea R et al. used the antibiotic protocol (amoxicillin (AMX) + metronidazole (MET)) for 102 patients for 12-month follow-up in nonsurgical periodontal therapy to obtain the maximum antimicrobial benefit and minimum risk for adverse effects. After comprehensive monitoring of the patients (ELISA and RT PCR for the detection of etiologic agents and inflammatory markers), the authors concluded that (AMX + MET) antibiotic protocols led to greater microbiological improvements compared to subgingival debridement alone [77].

Cha JK et al. also studied the clinical, microbial and radiographic effects of local minocycline combined with surgical treatment of peri-implantitis. The authors found that the repeated local delivery of minocycline combined with surgical treatment provides significant benefits in terms of clinical parameters and radiographic bone fill [78].

Luchian I et al. mentioned in a review that clindamycin offers several advantages for periodontal treatment, both systemically and locally, with various degrees of success, such as the enhancement of neutrophil chemotaxis, phagocytosis and the oxidative burst-oxidative stress storm, which are easily absorbed at the level of oral tissues in a considerable amount, substantial tissue penetration, especially inside the bone. All the above-mentioned features are synergistic, with a stimulating effect on the host immune system [56].

The management of periodontal diseases is an important issue. The American Dental Association Council on Scientific Affairs and the Center for Evidence-Based Dentistry conducted a systematic review and formulated clinical recommendations. The panel recommended against using antibiotics in most clinical scenarios, irrespective of definitive, conservative dental treatment availability. The experts suggested that antibiotics for target conditions should only be used when systemic involvement is present to avoid all the side effects of antibiotic therapy [79].

## 8. Conclusions

Periodontal disease is one of the most prevalent pathologies worldwide and it has the great advantage of being detectable in an early and efficient manner through RNA methods.

Using mRNA and circRNAs technologies, it was possible to identify new pathogenic mechanisms, new target genes and protective compounds, which lead to an improvement in the prognostic and may optimize future therapeutic protocols.

Therefore, RNA-based techniques can successfully detect periodontal bacteria much more accurately than others and they might represent a “state-of-the-art” diagnostic tool in the future.

The technology used for mRNA analysis should be standardized in the near future to be safely used by many clinicians in cooperation with molecular biology specialists as a useful tool for the early diagnosis of periodontitis.

## Figures and Tables

**Table 1:** Associations between periodontal disease and *Porphyromonas gingivalis* by mRNA analysis.

Authors Year, Country	Sample Type	mRNA Analysis Assay	Results	Novelty
Kantrong N et al., 2022, Khon Kaen, Thailand [23]	Gingival biopsies, healthy participants with periodontitis or clinically healthy gingiva	mRNA expressions/RT-qPCR of human β-defensin-2 (hBD-2), interleukin (IL-) 8, and IL-18 in stimulated GECs in the presence or absence of a caspase-4 inhibitor	mRNA upregulations of hBD-2, IL-8, and IL-18 upon *P. gingivalis* stimulation were significantly reduced by caspase-4 inhibition (*p* < 0.05), while, for *F. nucleatum*, the inhibitor did not exhibit the same suppressor activity	Caspase-4 activation in *P. gingivalis*-infected GECs showed an upregulation of immune effector molecules, suggesting a possible detection mechanism of caspase-4 in GECs in periodontal disease pathogenesis
Firatli Y et al., 2022, Turku, Finland [24]	Human gingival keratinocyte (HMK) monolayers were incubated with *P. gingivalis*, *F. nucleatum*, *P. gingivalis* LPS and IL-1β.	Immunoblots and mRNA levels by qPCR for protein levels of MCPIP-1 and MALT-1	MCPIP-1 mRNA levels were increased by *P. gingivalis*, *F. nucleatum*, and IL-1β, but no change was detected in MALT-1 mRNA levels	- Infection and inflammatory mediators regulate the gingival keratinocyte MCPIP-1 and MALT-1 mRNA and protein expression responses. - Periodontitis-associated bacteria-induced modifications in MCPIP-1 and MALT-1 responses can be a part of periodontal disease pathogenesis
Li D et al., 2022, Chongqing, China [25]	human gingival tissues	Dual-luciferase reporter assay, which assessed the binding of miR-185-5p to MZB1 (ER-localized protein)	MZB1 was markedly increased in the gingival tissues of periodontitis patients, in mouse models, and in the hPDLCs treated with lipopolysaccharide of *P. gingivalis* (LPS-PG)	MZB1 (a target gene of miR-185-5p) plays an important role in inhibiting the migration of hPDLCs through NF-κB signaling pathway and deteriorating alveolar bone loss
Wang H wr al., 2022, Shenyang, Liaoning Province, China [26]	the effects of the *P. gingivalis* outer membrane protein OmpH (encoded by PG0192 and PG0193) on IL-6 and tumor necrosis factor-α (TNF-α) expression in macrophages to assess the pro-inflammatory cytokine responses	Macrophages treated with mutant strains (PG0192-PG0193 deletion) showed a downregulation in the expression of IL-6 and TNF-α at mRNA and protein levels	IL-6 and TNF-α mRNA levels were up-regulated following treatment of macrophages with *P. gingivalis* W83 co-incubated with rOmpH-1 or rOmpH-2	- The roles of PG0192 and PG0193 in promoting IL-6 and TNF-α expression in macrophages exposed to *P. gingivalis* - Involvement of C5aR in the pro-inflammatory response
Bekić M et al., 2022, Belgrade, Serbia [27]	10 H-GMSC and 12 P-GMSC lines	*P. gingivalis* up-regulated the mRNA expression of IL-6, IL-8, MCP-1, GRO-α, RANTES, TLR-2, HIF-1α, OPG, MMP-3, SDF-1, HGF and IP-10 in P-GMSCs, while only IL-6, MCP-1 and GRO-α were up-regulated in H-GMSCs.	P-GMSCs had a significantly higher expression of MCP-1, RANTES, IP-10 and HGF compared to H-GMSCs, but IDO1 was lower	Cultures of P-GMSCs retain their pro-inflammatory properties, while exhibiting lower immunosuppressive potential than their healthy counterparts, and reduced regeneration-associated gene induction in culture. All these functions are positively influenced by *P. gingivalis* treatment
Huang XY et al., 2022, Fuzhou, China [28]	gingival tissue samples from clinically healthy subjects (15 cases) and patients with periodontitis (30 cases)	mRNA levels of the intracellular collagen triple helix repeat containing-1 (CTHRC1) and protein expression of the extracellular CTHRC1	In the periodontitis group, protein expression of CTHRC1 was higher than that of the clinically healthy group	- CTHRC1 might play a role in the development of periodontitis - Expression level might be significantly correlated with the stimulation produced by *P. gingivalis* LPS on fibroblasts
Braun ML et al., 2022, Vienna, Austria [29]	wild-type *T. forsythia* and *P. gingivalis* and T9SS signal peptidase-deficient mutants defective in protein secretion were used to stimulate human macrophages and gingival fibroblasts	mRNA expression levels of the pro-inflammatory mediators IL-6, IL-8, MCP-1 and TNF-α by qPCR	- 16 h post-stimulation, the *T. forsythia* T9SS mutant induced a significantly lower production of cytokines and the chemokine in cells compared to the corresponding wild-type strain - The opposite was noted for the *P. gingivalis* T9SS mutant	- T9SS shut-down translates into an altered inflammatory response towards periodontal pathogens - T9SS needs further evaluation as a potential novel target for periodontal therapy

**Table 2:** Associations between periodontal disease and *Aggregatibacter actinomycetemcomitans* by mRNA analysis.

Authors Year, Country	Sample Type	mRNA Analysis Assay	Results	Novelty
Jia R et al., 2022 Shanghai, China [30]	Gingival tissues of eighteen 8-week-old female rats were randomly distributed into three groups: Sham group, Trehalose group and *Lactobacillus helveticus* SBT2171 (LH2171) group	Expression of *β*-defensins, TNF-*α* IL-1*β* and IL-6 and the number of *A. actinomycetemcomitans* in rat gingival tissues by qRT PCR	mRNA level expression and release of inflammatory factors in the tissue samples in the LH2171 group were notably lower than those in the Trehalose group	*L. helveticus* improves alveolar bone resorption, increases the expression of β-defensins, inhibits the number *of A. actinomycetemcomitans* and prevents periodontitis
Wang B et al., 2021, Shaanxi, China [31]	- Mouse chronic periodontitis was induced by an in vivo ligature-induced periodontitis model - TPCA-1 (2-[(aminocarbonyl)amino]-5-(4-fluorophenyl)-3-thiophenecarboxamide (TPCA-1) is a IκB kinases (IKK) inhibitor) was intravenously injected into mice after chronic periodontitis induction	mRNA levels by qRT-PCR	- *A. actinomycetemcomitans* -induced expression of pro-inflammatory cytokines was inhibited in vitro by TPCA-1 treatment - NF-κB signal activation in osteoclasts	Treatment with TPCA-1 downregulates inflammation response and inhibits the osteoclastogenesis through - The inactivation of NF-κB pathway in chronic periodontitis mice model
Shiba F et al., 2021, Hyogo, Japan [32]	Effects of extracts from six different plants on Glycyrrhizin (GL)-suppressed TNF-α expression in *A. actinomycetemcomitans* -LPS-stimulated human oral keratinocytes (RT7)	At both mRNA and protein levels, *Equisetum arvense* (EA) extract had the strongest additive effect on the suppression of TNF-α by GL	LPS-induced periodontitis rat model showed that GL with EA supplementation significantly decreased TNF-α mRNA levels in the gingival tissue	GL and EA combination may improve the development of new oral hygiene products with the purpose of enhancing periodontal health
Pourhajibagher M et al., 2021, Tehran, Iran [33]	Curcumin-decorated nanophytosomes (Cur-NPhs) as a novel photo-sonosensitizer	Cur-NPhs-PSACT (photo-sonodynamic antimicrobial chemotherapy), the antimicrobial activities of Cur-NPhs against *A. actinomycetemcomitans* were investigated by analyzing cell viability, biofilm killing/degradation capacity, metabolic activity, expression of quorum-sensing-associated qseB and qseC genes, and biofilm-associated rcpA gene	The antimicrobial effect of Cur-NPhs-PSACT was dose-dependent	- Cur-NPhs-PSACT had antimicrobial activity against A. actinomycetemcomitans by decreasing the expression of virulence genes - Cur-NPhs attenuate this bacterium, potentially decreasing periodontal disease severity in patients
Kim MJ et al., 2020, Jellabukdo, Republic of Korea [34]	Anti-inflammatory effects of live oraCMU (Weissella cibaria CMU) against stimulation with the formalin-inactivated periodontal pathogen Aggregatibacter *actinomycetemcomitans* in RAW 264.7 macrophages	mRNA expression of proinflammatory cytokines such as IL1β and IL6 was assessed by qRT PCR	In *A. actinomycetemcomitans*-stimulated RAW 264.7 macrophages (Cell culture, The RAW 264.7 macrophage line (TIB-71, ATCC)), oraCMU reduced nitric oxide production by suppressing iNOS expression and downregulating the proinflammatory cytokines mRNA expression of in a dose-dependent manner.	Probiotic oraCMU showed anti-inflammatory activity associated with the inhibition of NF-κB signal activation in response to periodontal pathogens
Díaz-Zúñiga J et al., 2019, Santiago, Chile [35]	Effects of purified LPS, from serotypes a, b or c of *A. actinomycetemcomitans*, on primary cultures of microglia or mixed hippocampal cells	Cultures treated with serotype a-LPS displayed increased mRNA levels of the IL-4 and IL-10 modulatory cytokines	LPS from different *A. actinomycetemcomitans* serotypes triggers discriminatory immune responses, which differentially affect primary hippocampal cells	Serotype b-LPS treatment triggers the secretion of proinflammatory cytokines by microglia, induces neurite shrinking, and increases the extracellular Aβ1-42 levels, all features strongly associated with the etiology of Alzheimer’s disease.
Monasterio G et al., 2019, Santiago, Chile. [36]	Dendritic cells (DCs) play a central role in the host’s immune response during periodontitis; thus, this study aimed to analyze whether low-molecular-weight hyalurona (LMW-HA) has an immunostimulatory activity on DCs when stimulated with bacteria involved in periodontitis.	LMW-HA-treated and non-treated DCs were stimulated with *A. actinomycetemcomitans* or *P. gingivalis* and the mRNA expression for cytokines TNF- α, IL-1B, IL-6, and IL-23A was quantified by RT-qPCR	Higher expression levels of TNF-α, IL-1B, IL-6, and IL-23A were detected in DCs treated with LMW-HA after bacterial infection, as compared with non-treated DCs	LMW-HA plays an immunostimulatory role on the immune response triggered by DCs during infection with A. actinomycetemcomitans or *P. gingivalis*
Engström M et al., 2018, Stockholm, Sweden [37] Karolinska Institutet	Gingival tissue biopsy samples were obtained from 15 patients with periodontitis and 15 individuals with no periodontal disease. The presence of citrullinated proteins and expression of endogenous peptidylarginine deiminases (PAD2 and PAD4), in periodontal tissue of individuals with periodontitis and healthy controls, in relation to the periodontal pathogens *P. gingivalis* and *A. actinomycetemcomitans*	There was an increased staining of the citrullinating enzymes PAD2 and PAD4 in gingival connective tissue of patients with periodontitis, while similar levels of PAD2 and PAD4 were observed in the gingival epithelium of the two groups. Similarly, the mRNA expression of PADI2 and PADI4 were also increased in the gingival tissue samples of patients with periodontitis compared to the healthy group.	*P. gingivalis* and leukotoxins was comparable in both epithelium and connective tissue of the two groups	- Chronic gingival inflammation is associated with an increased local citrullination and PAD2 and PAD4 expression in periodontitis - The presence of *P. gingivalis* and *A. actinomycetemcomitans* leukotoxin were not connected, however, to the increased citrullination and PAD2 and PAD4 expression in periodontitis These two periodontitis pathogens have been suggested to be linked to anti-citrullinated protein antibodies in patients with rheumatoid arthritis

**Table 3:** Associations between periodontal disease and *Tannerella forsythia* by mRNA analysis.

Authors Year, Country	Sample Type	mRNA Analysis Assay	Results	Novelty
Braun ML et al., 2022, Vienna, Austria [29]	Wild-type *T. forsythia* and *P. gingivalis* and T9SS signal peptidase-deficient mutants defective in protein secretion were used to stimulate human macrophages and gingival fibroblasts	mRNA expression levels of the pro-inflammatory mediators IL-6, IL-8, MCP-1 and TNF-α by qPCR	T9SS shutdown translates into an altered inflammatory response in periodontal pathogens	T9SS as a potential novel target for periodontal therapy needs further evaluation
Yun IG et al., 2018, Gwangju, Korea [38]	The ability of *Litsea japonica* leaf extract (LJLE) to inhibit pro-inflammatory cytokine production in PDLFs in response to various stimulants	mRNA and protein expression	Anti-inflammatory effect of LJLE in PDLFs after infection with various oral bacteria, including *F. nucleatum*, *P. gingivalis*, *Treponema denticola*, and *T. forsythia*	LJLE has anti-inflammatory activity that could be exploited to control inflammation in human periodontitis
Eckert M et al., 2018, Bern, Switzerland [39]	Biofilm and gingival crevicular fluid (GCF)/ peri-implant sulcular fluid (PISF) samples were taken from 10 healthy tooth and implant sites, 12 gingivitis and mucositis sites, and 10 periodontitis and peri-implantitis sites	mRNA expression of individual genes	Gingipains’ expression level was associated with the levels of miropin and certain *T. forsythia* proteases around teeth but not implants	KLIKK-proteases, especially miropin, might be involved in the pathogenesis of both periodontal and peri-implant diseases
Lee SJ et al., 2017, Seoul, Korea [40]	The human oral epithelial cell line HOK-16B was infected *T. forsythia* and *F. nucleatum*, at various MOIs	RT-PCR and immunoblotting assays for mRNA and protein expression	Infection increased mRNA and protein expression of NLRP10, respectively NLRP10 is involved in activating the ERK signaling pathway in HOK-16B cells infected with *T. forsythia* and *F. nucleatum*	Pro-inflammatory cytokine IL-1α levels are augmented by the activation of the ERK pathway, which may play a critical role in periodontitis
Ran S et al., 2017, Shanghai, China [41]	Periapical lesions	mRNA levels of apoptosis-associated speck-like protein (ASC), caspase-1IL-1β, NLRP3 and AIM2 in THP-1-derived macrophages treated with *Porphyromonas* LPS were quantified by real-time PCR	Up-regulation of NLRP3 mRNA was correlated with a simultaneous up-regulation of caspase-1 mRNA in most samples	NLRP3 and AIM2 proteins are involved in the pathogenesis of periapical periodontitis. Anaerobes, such as *P. endodontalis*, *P. gingivalis*, *F. nucleatum* and *T. forsythia*, were the most important microbial stimuli that might activate inflammasomes in periapical tissues
Willi M et al., 2014, Zürich, Switzerland [42]	Gingival tissue	TREM-1 mRNA expression	TREM-1 expression was found to be increased in both aggressive and chronic periodontitis, compared to healthy tissues, and correlated with the levels of the ‘red complex’ species in the tissue	TREM-1 tissue expression is up-regulated in periodontal disease and correlates with the level of periodontal pathogens
